# A Case of Chronic Calcific Nonalcoholic Pancreatitis

**DOI:** 10.1155/2016/2963681

**Published:** 2016-11-10

**Authors:** Aaron Kangas-Dick, Umair Khan, Oluwafunbi Awoniyi, Shanza Waqar, Nu Nwe Tun, Karthikeyan Viswanathan, Cynthia Wong

**Affiliations:** ^1^School of Medicine, St. George's University, True Blue, Grenada; ^2^Department of Internal Medicine, Woodhull Medical Center, Brooklyn, NY, USA

## Abstract

Tropical Calcific Pancreatitis (TCP) is a type of chronic calcific nonalcoholic pancreatitis. Similar to nonalcoholic chronic pancreatitis, it presents in the second and third decades of life; however this type is reported mostly in the developing tropical and subtropical countries. It is associated with the formation of pancreatic calculi and a high probability of developing insulin-dependent diabetes mellitus. Epidemiologic studies have shown that these patients have an increased risk of developing pancreatic carcinoma. The etiology of TCP remains uncertain, with the current consensus suggesting genetics as well as possible toxicity from consuming large amounts of cassava, a tuber. Definite diagnosis of TCP requires younger age of onset, history of malnutrition, and presence of diabetes mellitus along with extensive pancreatic calcification and ductal calculi. When patients meet most but not all of these conditions the term Idiopathic Chronic Pancreatitis (ICP) is used. This is a case of a 44-year-old man who presented with most features seen in TCP, and however, was diagnosed with ICP.

## 1. Case Report

A 44-year-old male with a history of three previous hospitalizations for acute pancreatitis presented to the emergency department complaining of severe abdominal pain that radiated to the back with associated symptoms of nausea and vomiting.

The patient, originally from the Dominican Republic, experienced his first episode of acute pancreatitis at the age of 20. At that time, he was an occasional drinker. He moved to the United States 20 years ago, and was hospitalized for acute pancreatitis at our hospital in 2002. After this second episode, he reported that he quit drinking. He was admitted to another facility, again for pancreatitis, in 2011. He was admitted to our facility for a fourth time this year.

He had no significant medical or surgical history. There was no family history of any pancreatic diseases. He denied taking any medications, including herbal supplements. He works as a taxi driver and denied the use of tobacco products and recreational drugs.

On physical exam he had epigastric tenderness as well as tenderness in the right upper and left upper quadrants of the abdomen with mild guarding.

His lipase level was 1175 U/L and amylase was 554 U/L and consistent with an episode of acute pancreatitis. Serum tests confirmed the absence of alcohol and a drug test was negative. HbA1C was 6.1%.

An abdominal CT done during this admission demonstrated a homogenous pancreatic parenchyma without any pancreatic necrosis. Calcifications were associated with the neck, head, and uncinate process. These calcifications were also seen in the abdomen CT that was done back in 2002. An ultrasound of the gallbladder performed during this admission showed no gallstones and no significant dilatation of the bile ducts.

A genetic study was done and he was found to have no variations in the SPINK1 gene. No other genetic testing was done during this admission.

He was diagnosed with Idiopathic Calcific Pancreatitis and treated symptomatically.

## 2. Discussion

Some patients have recurrent episodes of pancreatitis due to an unknown or yet to be determined etiology and are diagnosed with Idiopathic Chronic Pancreatitis (ICP). Here we highlight the overlap along with the differences between ICP, TCP, and Fibrocalculous Pancreatic Diabetes (FCPD), with an emphasis on TCP.

TCP is relatively rare in the developed world. It occurs most often in the second and third decades of life [1]. It is associated with the development of insulin-dependent diabetes mellitus in approximately 90% of patients, often before the age of 30 [2, 3]. When diabetes is present, the condition may also be referred to as FCPD [1]. It is associated with an increased lifetime risk of pancreatic cancer when compared with patients with other forms of chronic pancreatitis [3, 4].

Tropical Calcific Pancreatitis (TCP) was first brought to attention in a report by Zuidema among young diabetics in Indonesia, in whom he noticed fibrosis and calcification of the pancreas [5]. It is now being increasingly recognized as a cause of early onset nonalcoholic chronic pancreatic disease in parts of the developing tropical and subtropical world [6]. Cassava, a tuber, is consumed as a staple food in these developing tropical parts of the world by people of lower socioeconomic status [5]. It contains cyanogenic glycosides such as linamarin and lotaustralin, which when ingested can only be removed from the body via sulfur containing compound [5]. As malnourished individuals are usually deficient in sulfur containing amino acids the cyanogen glycosides accumulate and cause oxidative stress and free radical injury. Hence, the association of cassava as a cotrigger for TCP [5]. However, TCP has been reported in populations where cassava is not consumed [5], and prolonged cassava consumption did not produce pancreatitis in the rat model [7]. It has also been reported in patients with no history of cassava in their diets, from higher socioeconomic groups, and in familial clusters suggesting other contributing factors [2].

Genetic etiologies such as a mutation in the serine protease inhibitor Kazal type 1 (SPINK1) gene have been reported in the progression of TCP [6]. Alterations in the genes SPINK1 and Chymotrypsinogen C are highly associated with TCP [2]. Others include mutations in the Cathepsin B, Carboxypeptidase A1, CFTR, Glycoprotein 2, transcription factor 7-like 2, and calcium sensing receptor gene [5].

The etiology remains unclear with a range of factors including malnutrition, cassava consumption (cyanogen toxicity), genetic factors, oxidative stress, free radical injury, and trace element deficiency, all playing a role in the pathogenesis [5].

The clinical presentation resembles an acute pancreatitis episode. Features of TCP seen on imaging include large intraductal calculi and pancreatic atrophy [3, 6]. Some patients progress to insulin-dependent, ketosis-resistant, diabetes mellitus [6]. To be diagnosed with TCP, patients must meet the following, distinctive yet arbitrary, criteria: onset at less than thirty years of age, BMI less than 18 kg/m^2^, absence of any other cause of pancreatitis, and the presence of diabetes [3, 5]. Since these are arbitrary criteria, cases of TCP are now being diagnosed in relatively older populations and only a fraction of the cases satisfy the criteria of classical TCP [5]. This is justified because a low BMI does not necessarily mean being malnourished. Moreover, cases have also been diagnosed in patients with normal body weights as well as in obese individuals, and malnourishment was not always present [1]. Also insulin-dependent diabetes mellitus is an end-point, and not always present earlier in the course of the disease [5].

But definite diagnosis of TCP requires younger age of onset, history of malnutrition, and presence of diabetes mellitus along with extensive pancreatic calcification and ductal calculi [8].

When patients do not meet the criteria for Tropical Calcific Pancreatitis, they are ultimately diagnosed with Idiopathic Chronic Pancreatitis (ICP) [8]. Approximately 40% of diagnoses are considered idiopathic [9].

In our case the patient had no risk factors for pancreatitis such as chronic alcohol intake, gallstones, or hypertriglyceridemia. His first episode of pancreatitis was at the age of twenty and since then he has had three more episodes. Imaging done during this admission and during previous admission demonstrate calcifications in the pancreas. Hence, due to the absence of any other risk factors for pancreatitis, based on his history, clinical presentation, absence of any variations in SPINK1 gene, and minimal pancreatic calcifications on imaging findings, he was diagnosed with ICP and treated symptomatically. ICP, thus, is a diagnosis of exclusion [10].

If he were to go on to develop diabetes mellitus and extensive pancreatic calcifications, a diagnosis of TCP can then be definitely made.

Management of patients with TCP includes medical management and surgery when abdominal pain is intractable. Surgical options include ductal decompression and drainage techniques. In addition to relief from pain, improvement in diabetes has been reported after surgical intervention [11].

Thus it is vital for clinicians to consider a diagnosis of ICP or TCP in patients presenting with signs and symptoms of chronic pancreatitis in the absence of gallstones, ethanol, toxic exposure, or a family history. These patients should receive a thorough evaluation of pancreatic function as well as imaging studies to determine the extent of pancreatic calcification and to establish the extent of pancreatic damage.

In addition there is a significant cost to the healthcare system due to chronic pancreatitis. In 2009, among gastrointestinal and hepatology principal discharge diagnoses made in the US, 19,724 were chronic pancreatitis, with an aggregate cost of approximately $172m [12].
